# Personalizing Value in Cancer Care: The Case for Incorporating Patient Preferences Into Routine Clinical Decision Making

**DOI:** 10.2196/13800

**Published:** 2019-07-10

**Authors:** Joshua Seidman, Domitilla Masi, Amalia Elvira Gomez-Rexrode

**Affiliations:** 1 Avalere Health Center for Healthcare Transformation Washington, DC United States; 2 University of Michigan Medical School Ann Arbor, MI United States

**Keywords:** shared decision making, patient preference, health care, neoplasms

## Abstract

Despite growing research demonstrating the potential for shared decision making (SDM) to improve health outcomes, patient preferences—including financial trade-offs—are still not routinely incorporated into health care decision making. As the US health care delivery system transitions to rewarding value-based care, the question of “value to whom?” assumes greater importance. To achieve the goals of value-based care, the patient voice must be incorporated into clinical decision making by embedding SDM as a routine part of clinical practice. Identified as a priority by the Centers for Medicare & Medicaid Services (CMS), SDM-related measures and initiatives have already been integrated into CMS’ Center for Medicare and Medicaid Innovation (Innovation Center) demonstration projects (eg, the Oncology Care Model and Transforming Clinical Practice Initiative) and value-based payment programs (eg, the Merit-based Incentive Payment System, Medicare Shared Savings Program) to incentivize more proactive SDM engagement between patients and their providers. Furthermore, CMS has also integrated formal shared decision-making encounters into coverage and reimbursement policies (eg, for implantable cardioverter defibrillators), demonstrating a growing interest in SDM and its potential for eliciting and promoting the integration of patient preferences into the clinical decision-making process. In addition to increasing policy efforts to promote SDM, we need more research investments aimed at understanding how to optimize the science and practice of meaningful SDM. The current landscape and proposed road map for next steps in research, outlined in this review article, will help ensure the transition of pilots and research projects regarding the implementation of SDM into sustainable solutions.

## Introduction

### The Need for More Personalized Cancer Care

In the race toward an emerging health care system that rewards providers for the value they provide, the question of “value to whom?” holds great significance. Ultimately, we should measure the impact of the care delivered by health care providers on the basis of improvement in outcomes that matter to their patients relative to the costs of achieving those results. Patients will not accept a *value-based care* system if it rewards providers for outcomes that the patients did not seek when they embarked on their care journeys. Therefore, the success of value-based payment models requires that providers incorporate patient preferences into routine clinical decision making. Virtually all assessment of value, however, presumes a set of benefits that remains constant from one patient to the next, failing to account for how individualized preferences shape the value equation. In fact, decades of research demonstrate that no singular correct answer exists for many health care decisions because so many of them involve a series of trade-offs. The side effect of neuropathy may have a profoundly different impact on a concert pianist than a theoretical physicist.

The transition to new care delivery and payment models provides incentives for providers to change behavior. Those models are gradually encouraging more attention to meeting patients’ diverse needs and preferences, but greater opportunity exists for us to shape that future, especially in the care of people with cancer. This paper provides context for the need to better incorporate patient preferences into routine clinical decision making, presents an analysis of the policy landscape and a business imperative for doing so, and proposes a road map for next steps in research.

### The Promise of SDM in Personalizing Cancer Care

Shared decision making (SDM) is “a process of communication in which clinicians and patients work together to make optimal healthcare decisions that align with what matters most to patients” [1,2]. The process of SDM requires that patients and clinicians discuss potential health care options within the context of a patient’s preferences, broadly defined as their values, needs, goals, and expectations, including trade-offs related to the direct and indirect costs of care. Research demonstrates how SDM contributes to better care and improved patient experiences. Research has also shown that SDM and the incorporation of patient preferences into clinical decision making have the potential to improve key measures of health care quality that matter to patients and drive more appropriate health care utilization [3]. Moreover, patients greatly appreciate clinicians who acknowledge their individual needs, which is important given that the patient-provider relationship can impact patients’ health care outcomes such as treatment adherence [4].

However, the routine incorporation of patient preferences into clinical practice and decision making remains limited [4]. Research led by the Patient Advocate Foundation (PAF) shows that cancer patients’ health care–related preferences vary greatly among individuals based on the progression of the disease, stage of life, and demographics [4]. Qualitative research conducted by Cancer *Care* found that cancer patients want their providers to understand and appreciate the effect treatment has on their lives, such as on their jobs, financial status, family and household responsibilities, access to transportation, and significant upcoming plans [5]. However, a survey with 3000 American adults with cancer found that patients do not receive the information they need to make health care decisions based on their personal preferences and values [6].

Research also shows that cancer patients are at high risk for financial hardship, and financial trade-offs are an increasingly important part of decision making in oncology care [7]. For example, in a 2018 survey conducted by PAF with 1158 patients, most respondents noted that their financial hardship is a direct result of out-of-pocket medical costs such as coinsurance, copays, and deductibles. A third of PAF’s survey respondents with cancer reported that their financial hardship also stemmed from costs related to time off work, childcare, and transportation [8]. Data suggest that nearly one-third of cancer patients report making changes to their prescription drug use, such as skipping doses and delaying filling a prescription, for financial reasons [9]. Despite the prevalence of financial distress and the impact it has on patients’ quality of life and outcomes, treatment costs and patients’ financial distress are rarely discussed by oncology clinicians [10]. In Cancer *Care* ’s survey, less than half of the respondents reported having adequate information on whether they would be able to work, the care they would need at home, and the out-of-pocket cost of treatment [6]. In PAF’s 2018 survey, only 30% of respondents with cancer noted that their providers explained the costs of their treatment options [8].

As we drive toward the promotion of SDM as a means to incorporate the patient voice into clinical decision making, further research is required to optimize the practice and science of SDM and ensure that patients’ personal goals, needs, values, and expectations guide the decision-making process.

## Discussion

### Analysis: Policy Landscape and Business Imperative for Implementing Meaningful Shared Decision Making

The changing US payment environment provides direct and indirect incentives for providers to engage proactively in SDM. The Centers for Medicare & Medicaid Services (CMS) has explicitly identified SDM as a priority and has experimented with direct SDM reimbursement through a Center for Medicare and Medicaid Innovation (Innovation Center) demonstration. CMS has also created a set of requirements and expectations in some of its alternative payment models (APMs). CMS continues to develop performance measures related to SDM to drive more proactive SDM engagement among providers through its Quality Payment Program (QPP) that relies on an evolving set of performance measures and clinical practice improvement activities. Indirectly, CMS has identified SDM as a critical component in APMs and other value-based payment arrangements, encouraging providers to use SDM to succeed in achieving high-quality, cost-effective care that most appropriately meets beneficiaries’ expectations. Medicare is a federal health insurance program for individuals who are aged 65 years or older, certain younger individuals with disabilities, and those with end-stage renal disease. Medicaid is a joint federal and state program that provides medical cost assistance and coverage to some low-income and under-resourced individuals. Specifications of Medicaid programs vary by state, and some individuals are able to qualify and receive coverage under both Medicare and Medicaid programs [11].

#### Specific Innovation Center Demonstration Programs That Encourage Shared Decision Making

Table 1 summarizes the various programs encouraging SDM.

CMS promotes SDM through the Innovation Center. For example, the Innovation Center launched the Comprehensive Primary Care (CPC) Initiative, a 4-year multipayer initiative designed to strengthen primary care. CPC serves as the foundation for the Comprehensive Primary Care Plus (CPC+), a 5-year advanced primary care medical home model. Fundamental to this initiative and the practice redesign model are 5 CPC functions, including “Patient and Caregiver Engagement,” which promotes the integration of decision aids for preference-sensitive conditions into routine care for primary care practices [12].

**Table 1:** Summary of Innovation Center demonstration programs encouraging shared decision making.

Model, program or policy	Description	Patient population	Participants	Status
Comprehensive Primary Care Plus (CPC+) [12]	A national advanced primary care medical home model aiming to strengthen primary care through regionally based multipayer payment reform and care delivery transformation	Medicare fee-for-service (FFS) beneficiaries	2900 primary care practices across 18 regions	Ongoing
Oncology Care Model (OCM) [13]	A specialty care payment and delivery model aiming to leverage financial incentives to encourage improved care coordination, appropriateness of care, and access to care for beneficiaries undergoing chemotherapy	Medicare FFS beneficiaries receiving chemotherapy treatment	179 practices and 13 payers	Ongoing
Transforming Clinical Practice Initiative (TCPI) [14]	A primary care transformation model aiming to leverage peer-based learning networks to support over 140,000 clinicians with large-scale health transformation and comprehensive quality improvement strategies	Medicare and Medicaid beneficiaries, and commercial patients who visit TCPI primary and specialty care clinics	29 practice transformation networks and 12 support and alignment networks	Ongoing
Beneficiary Engagement and Incentives Models [15,16]	A set of 2 models aiming to increase patient engagement in the care decision-making process by testing varying approaches to shared decision making (SDM)	Medicare FFS beneficiaries	SDM model: Practitioners participating in accountable care organizations; Direct Decision Support model: Decision Support Organizations operating outside of the clinical delivery system	Cancelled

The Oncology Care Model (OCM)—which aims to provide high-quality and coordinated oncology care through an episode payment model—also promotes SDM by holding providers accountable for documenting a care plan. OCM specifies that the care plan must address the 13 components of the Institute of Medicine’s Care Management Plan for each participating beneficiary. The components include aspects of a patient’s care closely related to their preferences and goals, such as the following:

- Treatment goals (curative, life-prolonging, symptom control, and palliative care)
- Treatment benefits and harms, including common and rare toxicities and how to manage these toxicities, as well as short-term and late effects of treatment
- Information on quality of life and a patient’s likely experience with treatment
- Estimated total and out-of-pocket costs of cancer treatment
- A plan for addressing a patient’s psychosocial health needs, including psychological, vocational, disability, legal, or financial concerns and their management

The Transforming Clinical Practice Initiative (TCPI) supports integration of SDM into the clinical workflow by supporting over 140,000 clinician practices across the United States in developing and sharing their quality improvement strategies to achieve large-scale health care transformation [14]. For example, Mayo Clinic, a practice transformation network awardee, aims to engage 1231 clinicians in a proposed care delivery model that integrates SDM as a quality improvement strategy to better engage patients in their treatment decisions [17,18]. Mayo Clinic maintains an SDM National Resource Center, which aims to advance patient-centered medical care by providing resources, toolkits, and decision aids to caregivers in both primary and specialty care [19]. In addition, the Iowa Healthcare Collaborative (Compass) practice transformation network supports more than 7000 clinicians across 6 states and partners with designated clinical and operational leads and quality improvement advisors for every practice in each state. Compass practice transformation network quality improvement advisors work closely with clinical practices on SDM best practices to focus on integrating patients and their families as equal partners in their health and health care. SDM is 1 of 6 TCPI Person and Family Engagement (PFE) metrics that align with CMS’ PFE strategy to strengthen persons and families as partners in their care while achieving the overarching CMS strategy.

In 2016, the Innovation Center announced the Beneficiary Engagement and Incentives models, which included 2 new models focused on SDM: (1) the SDM Model focused on integrating a 4-step SDM process into routine clinical practice of accountable care organizations (ACOs) to better inform and engage beneficiaries [15] and (2) the Direct Decision Support (DDS) Model, which aimed to test an approach to SDM delivered outside of the clinical delivery system by health management and decision support services organizations [16]. The Innovation Center’s announcement of these models further reflects how CMS views SDM as a critical component to improve the cost, quality, and experience for Medicare beneficiaries.

CMS, however, announced the cancellation of the SDM Model in 2017 followed by the DDS Model in the first half of 2018 as a result of technical issues and apparent lack of interest from qualifying ACOs. This cancellation signals there is still a long way to go in understanding how to appropriately hold providers accountable for SDM and construct models that integrate SDM into their existing workflows.

As the Innovation Center continues to struggle with the best vehicles for achieving SDM accountability, CMS released a request for information in September 2017 for a new direction for the Innovation Center. The request for information focuses on promoting patient-centered care and testing new market-driven reforms that support SDM and engagement efforts by empowering beneficiaries as consumers and improving price transparency [20]. Issuing this request for information demonstrates CMS’ continued interest in beneficiary engagement and signals that CMS will continue to push toward elevating SDM.

#### Centers for Medicare & Medicaid Services’ Promotion of Shared Decision Making Accountability via Performance Measurement

Table 2 summarizes the measures promoting SDM.

CMS has also integrated SDM-related quality measures into several value-based payment programs, including the Merit-based Incentive Payment System (MIPS), the FY 2019 Medicare Hospital Inpatient Prospective Payment Systems (IPPS) Rule, and the Medicare Shared Savings Program (MSSP).

Established by the QPP, MIPS is a value-based payment pathway for clinicians billing under Medicare part B, which broadly rewards value and outcomes. Most clinicians who are subject to the QPP are expected to participate in the MIPS pathway. For calendar year 2019, CMS estimates that 650,165 clinicians will be eligible to participate in MIPS [22].

CMS calculates an MIPS score and associated payment adjustment based on 4 performance categories, 1 of which involves practice improvement activities, which comprises 15% of the final score for the 2019 reporting year. Providers select from an inventory of activities that include several related to SDM and beneficiary engagement [21]:

- Engagement of patients, family, and caregivers in developing a plan of care
- Improved practices that engage patients previsit
- Use of evidence-based decision aids to support SDM

Another MIPS performance category focuses on quality measures, which comprises 45% of the final score for the 2019 reporting year. There are 2 SDM-related measures for the 2018 performance year [21]:

- Total knee replacement—SDM: trial of conservative (nonsurgical) therapy
- Hepatitis C—discussion and SDM surrounding treatment options

On August 2, 2018, CMS released the FY 2019 IPPS final rule, which outlines CMS’ perspective and response to public comments on inclusion of 1 SDM-related process measure (NQF #2962) [23] in the Prospective Payment System-Exempt Cancer Hospital Quality Reporting program [22]. Specifically, the measure would assess patient-reported outcomes on the SDM processes to have surgery. In addition, the measure would assess the extent to which health care providers actually involve patients in a decision-making process that includes the essential elements of SDM such as laying out options, discussing the reasons to have the intervention and not to have the intervention, and asking for patient input [23].

CMS’ policy changes to include SDM-related measures in value-based payment models demonstrate an increased commitment to incentivizing SDM and the incorporation of patient preferences into clinical decision making. However, fully assessing the extent to which patient and family preferences have been adequately elicited and integrated will require the development and use of more robust measures to assess the decision-making process of clinicians and beneficiaries.

**Table 2:** Summary of Centers for Medicare & Medicaid Services’ measures promoting shared decision making accountability.

Program or rule	Description	Patient population	Example SDM^a^ measures
Merit-based Incentive Payment System [21]	A value-based payment pathway, established by the Quality Payment Program, for clinicians billing under Medicare part B. The program broadly rewards value and outcomes by measuring clinician performance in 4 areas: quality, improvement activities, promoting interoperability, and cost	Medicare beneficiaries	Total Knee Replacement—SDM: trial of conservative (nonsurgical) therapy; Hepatitis C-discussion and SDM surrounding treatment options
Financial year 2019 Medicare Hospital Inpatient Prospective Payment System rule [22]	The final rule implements adjustments to the inpatient payment system	Medicare fee-for-service (FFS) beneficiaries	SDM Process (NQF #2962) [23]
Medicare Shared Savings Program [24]	An alternative payment models that encourages physicians, hospitals, and other health care providers to form accountable care organizations (ACO) to provide coordinated and high-quality care to their patients. Participants can select between 4 tracks with varying financial risk arrangements	Medicare FFS beneficiaries	Consumer Assessment of Healthcare Providers and Systems (CAHPS): how well your providers communicate (ACO measure 2); CAHPS: health promotion and education (ACO measure 5); CAHPS: SDM (ACO measure 6); CAHPS: stewardship of patient resources (ACO measure 34) [25]

^a^SDM: shared decision making.

Another approach to deploying performance measurement as a lever to drive SDM involves the more broadly focused member experience measures, given the evidence linking SDM to greater patient satisfaction [26]. The MSSP encourages providers to form an ACO to provide coordinated and high-quality care to their Medicare patients. The program provides ACOs with various participation options (or tracks) depending on the financial risk they choose to assume [24]. In the 2018 performance year of the MSSP, ACOs are evaluated through the assessment of 31 quality measures. Of these, 4 measures (within the patient/caregiver experience domain) are Consumer Assessment of Healthcare Providers and Systems measures related to SDM and the integration of preferences into clinical decision making ([25]. These measures assess the following:

- How well providers communicate
- Health promotion and education
- SDM
- Stewardship of patient resources (ie, care team talked with patient about cost of their prescription medicines) [27]

There also exist several SDM-related measures that have been validated by their developers but not yet integrated into Medicare reporting and/or value-based programs. Although not all measures are developed with the intention for integration into Medicare programs, there still exists an opportunity for stakeholders to advocate for inclusion of these validated measures into CMS’ quality programs. For example, such measures include the American Academy of Neurology’s “Patient/Caregiver Nutritional Preferences” [28] and “Patient Counseled About Advanced Health Care Decision-Making, Palliative Care, or End-Of-Life Issues,” [29] the American College of Cardiology/American Heart Association’s “SDM Between Physician and Patient in Anticoagulation Prescription Prior to Discharge” [30] and “SDM Between Physician and Patient in Anticoagulation Prescription,” [30] and the Society for Interventional Radiology’s “Communication and SDM with Patients and Families for Interventional Oncology Procedures” [31].

#### Future Directions for Centers for Medicare & Medicaid Services Policies Designed to Drive Shared Decision Making

More recently, CMS has also taken steps to integrate SDM into a coverage and reimbursement policy. For example, CMS finalized changes to the implantable cardioverter defibrillator (ICD) national coverage determination (NCD). Specifically, the update requires a formal SDM encounter between the patient and a physician or qualified nonphysician practitioner (eg, physician assistant or nurse practitioner) before ICD implantation for certain patient populations [32]. This requirement demonstrates that CMS is continuing to promote the integration of patient preferences into the clinical decision-making process. This also suggests that CMS may develop new requirements around SDM and patient engagement into coverage and reimbursement policy.

In August 2018, an advisory panel to CMS strongly recommended that CMS integrate patient-reported outcomes into future NCDs for Chimeric Antigen Receptor T-cell (CAR-T) therapies. The Medicare Evidence Development & Coverage Advisory Committee argued that coverage of CAR-T therapies should take into consideration a variety of patient-reported outcomes including assessments of how the clinical decisions account for individual patient preferences.

The direction that CMS is taking to promote and incentivize greater SDM between patients and clinicians poses an opportunity for providers to engage in the practice of SDM and position themselves for greater success across an increasing number of value-based payment models. The increasing integration and promotion of SDM in CMS programs suggests that SDM has the potential to play a critical role in improving providers’ performance in value-based programs. However, this would require timely development and testing of strategies and tools to support meaningful SDM as well as more robust approaches to SDM payment models and provider accountability.

### Road Map: Next Steps in Research

As the policy environment begins to shift toward promoting a greater focus on placing the patient and family at the center of the care decision-making process, 3 key opportunities exist.

First, we need to improve patient and provider engagement in SDM, that is, we need further research on appropriate tactics, approaches, and resources to better prepare patients and families to share their preferences (including financial trade-offs) during clinical decision making. We also need best practices for educating and training providers on how to engage in SDM, so they can successfully elicit and integrate patient preferences in care decision making.

Second, successful payment models will require the development and use of robust SDM performance measures. These measures need to assess whether a successful SDM encounter took place as well as its impact on key outcomes of interest, including patient satisfaction with care. In addition, given that some validated SDM measures have been developed but not yet integrated into Medicare quality programs, an opportunity exists for stakeholders to advocate for their inclusion in these programs. Value-based payment models, if well designed, over the long term will encourage SDM. But in the short-term, there may be a need to think about alternatives to promote SDM given the upfront investment of time, resources, and technology costs that are required to achieve meaningful SDM. Research is needed to assess how to ensure that we are not creating unnecessary burdens.

Third, to succeed, SDM needs to be respectful of the demands that already exist on providers as well as on patients and caregivers. Further research should explore how to integrate SDM into the clinical workflow in ways that minimize provider burden and meet patients where they are. This will require consideration of the role of the multidisciplinary team and ideal points in patient’s care journey where SDM should be implemented. This will also require the development of resources, tools, and technologies (both patient- and provider-facing tools). For example, health information technology should be used to create new real-world evidence and better incorporate preferences into clinical decision making through dashboards. Such dashboards could pull information from patients and better capture and exchange information from providers’ clinical electronic data.

Finally, patient contribution and participation should be built into the fabric of improving SDM tools, measures, resources and processes. That is, patients should be a key stakeholder included in and central to the development of solutions by, for example, using human-centered design principles to include the patients’ perspective in all aspects of the development of such, and ensuring that patients have a seat on advisory boards and other committees that guide this work.

## Conclusions

Despite research showing that SDM has the potential to improve health outcomes, patient preferences—including those regarding financial trade-offs—are still not routinely incorporated into health care decision making. Given the evidence that demonstrates how SDM can support more effective value-based care, the transition to an increasingly value-based payment environment has great potential to drive the routinization of SDM in standard care delivery over the long term. In the more immediate time frame, direct and indirect incentives for providers to engage in SDM, embedded in payment and delivery reform space, show promise for integrating patient preferences into the heart of clinical decision making. Moreover, the proposed road map for next steps in research will help ensure the transition of pilots and research projects regarding the implementation of SDM into sustainable solutions.

## Abbreviations

ACO: accountable care organization
APM: alternative payment model
CAR-T: Chimeric Antigen Receptor T-cell
CMS: Centers for Medicare & Medicaid Services
CPC: Comprehensive Primary Care
CPC+: Comprehensive Primary Care Plus
DDS: Direct Decision Support
ICD: implantable cardioverter defibrillator
IPPS: Inpatient Prospective Payment System
MIPS: Merit-based Incentive Payment System
MSSP: Medicare Shared Savings Program
NCD: national coverage determination
PAF: Patient Advocate Foundation
PFE: Person and Family Engagement
QPP: Quality Payment Program
SDM: shared decision making
TCPI: Transforming Clinical Practice Initiative
